# Interprofessional collaboration among health professionals in cleft lip and palate treatment and care in the public health sector of South Africa

**DOI:** 10.1186/s12960-021-00566-3

**Published:** 2021-02-27

**Authors:** Phumzile Hlongwa, Laetitia C. Rispel

**Affiliations:** 1 School of Oral Health Sciences, Faculty of Health Sciences, University of the Witwatersrand, Johannesburg, South Africa; 2 School of Public Health, Faculty of Health Sciences, University of the Witwatersrand, Johannesburg, South Africa; 3 Centre for Health Policy & SARChI Chair, School of Public Health, Faculty of Health Sciences, University of the Witwatersrand, Johannesburg, South Africa

**Keywords:** Interprofessional collaboration, Cleft lip and palate, Multi-disciplinary, Health professional, South Africa

## Abstract

**Background:**

Collaboration among different categories of health professionals is essential for quality patient care, especially for individuals with cleft lip and palate (CLP). This study examined interprofessional collaboration (IPC) among health professionals in all CLP specialised centres in South Africa’s public health sector.

**Methods:**

During 2017, a survey was conducted among health professionals at all the specialised CLP centres in South Africa’s public health sector. Following informed consent, each member of the CLP team completed a self-administered questionnaire on IPC, using the Interprofessional Competency Framework Self-Assessment Tool. The IPC questionnaire consists of seven domains with 51 items: care expertise (8 items); shared power (4 items); collaborative leadership (10 items); shared decision-making (2 items); optimising professional role and scope (10 items); effective group function (9 items); and competent communication (8 items). STATA^®^13 was used to analyse the data. Descriptive analysis of participants and overall mean scores were computed for each domain and analysed using ANOVA. All statistical tests were conducted at 5% significance level.

**Results:**

We obtained an 87% response rate, and 52 participants completed the questionnaire. The majority of participants were female 52% (*n* = 27); with a mean age of 41.9 years (range 22–72). Plastic surgeons accounted for 38.5% of all study participants, followed by speech therapists (23.1%), and professional nurses (9.6%). The lowest mean score of 2.55 was obtained for effective group function (SD + -0.50), and the highest mean score of 2.92 for care expertise (SD + -0.37). Explanatory factor analysis showed that gender did not influence IPC, but category of health professional predicted scores on the five categories of shared power (*p* = 0.01), collaborative leadership (*p* = 0.04), optimising professional role and scope (*p* = 0.03), effective group function (*p* = 0.01) and effective communication (*p* = 0.04).

**Conclusion:**

The seven IPC categories could be used as a guide to develop specific strategies to enhance IPC among CLP teams. Institutional support and leadership combined with patient-centred, continuing professional development in multi-disciplinary meetings will also enrich IPC.

## Introduction

The global discourse on interprofessional collaboration (IPC) or the ability of health professionals to collaborate or work together as a team has intensified [1–7]. IPC is defined as: “*multiple health workers from different professional backgrounds working together with patients, families, caregivers, and communities to deliver the highest quality of care*” [5]: p. 13. The envisaged benefits of IPC include identifying and drawing on the strengths of each member of the health professional team and using those strengths to prevent and manage complex diseases, provide quality of care, and improve both patient and health worker outcomes [5, 6]. This is because IPC improves communication and teamwork and promotes coordination across the continuum of health care [5, 6]. IPC also facilitates egalitarian relationships among health professionals [8], and assists with the amelioration of health workforce shortages [5]. Some scholars suggest that the lack of or sub-optimal IPC among members of health-care teams contributes to poor health-care quality [9].

Research on IPC indicates that patient outcomes and quality of care are enhanced and costs are reduced when health-care team members work together towards shared patient-centred goals [9–16]. IPC has been reported to benefit patients with non-communicable diseases and mental disorders [9]. A study that evaluated the effect of pharmacist participation in medical rounds in an intensive care unit demonstrated a two-thirds reduction in preventable adverse drug events due to prescribing errors [17]. A systematic review of 36 randomised controlled trials involving IPC demonstrated that the risk of hospital readmission was reduced by 19%, while emergency department visits among older adults was reduced by 31% [18]. A study found that patients treated by IPC teams were more satisfied with the care they received [19]. Another study in an acute care setting found that IPC resulted in a decrease in readmissions and an overall decrease in catheter-associated urinary tract infections over time [3].

IPC has also been found to benefit health professionals in primary health-care settings [20], while studies on IPC in palliative care and geriatric care demonstrated mutual benefits for patients and members of the health-care team [19, 21].

A number of competency frameworks have been developed to assess IPC among health professionals in different settings [22–27]. These frameworks evaluate various attributes of IPC such as communication, care coordination, decision-making, power imbalances, role expectations, teamwork, shared responsibility, and organisational culture. The frameworks differ in the number of domains and/or assessment items, the study setting, development methodology, and measurement scales [22–27]. These competency frameworks have been criticised for limiting innovation and interfering with interprofessional practice [28], variations in quality since they were designed for specific populations and purposes, and insufficient or lack of validation in different geographical settings [29]. Nonetheless, the frameworks are useful in generating empirical information on IPC and in pointing to areas for improvements.

The World Health Organization (WHO) has pointed out that IPC by itself will not achieve the desired outcomes, but requires a set of enablers [5]. These IPC enablers include visionary leadership, institutional support, mentorship and learning, and positive practice environments [5, 6, 8]. A multi-country case study on IPC in primary health care in Brazil, Canada, India, South Africa and the United States of America (USA) recommended similar enablers or success factors, as well as supportive legislation for the health and education sectors, dedicated funding and other resources, and strong linkages between academia and clinical care [6].

Research studies have also identified various barriers to IPC, such as professional cultures and stereotypes, often created by the process of professional training and socialisation [6]. Other barriers to IPC include silo practice in many health-care settings, curricula and accreditation requirements of health professions’ regulatory authorities and inadequate knowledge of the roles and scopes of different health professions [6, 8, 30].

Individuals born with cleft lip and palate (CLP), a complex craniofacial congenital anomaly, require treatment and care from birth until early adulthood by a multi-disciplinary team (MDT) of health professionals with specialised skills and expertise [31, 32]. These MDT members include inter alia, plastic surgeons, maxillo-facial surgeons, orthodontists, paediatricians, psychologists, professional nurses, social workers, speech therapists and audiologists [33–35].

Studies in high-income countries have found that IPC is a major contributor to the success in the treatment and care of individuals with CLP [36, 37]. However, these studies have not measured IPC across the domains of care expertise, collaborative leadership, shared power, effective group function, optimising professional role and scope, effective communication.

In many African countries, including South Africa, IPC remains a fairly new concept [38, 39]. There has been an increasing calls for interprofessional education in South Africa to facilitate IPC [38] and universal health coverage reforms [40]. We could not find any studies in South Africa that have focused on IPC in the context of CLP care or that have measured IPC using a validated competency framework. Hence, the aim of this study was to measure IPC among health professionals at all specialised CLP treatment centres in South Africa’s public health sector. This IPC study was part of a larger doctoral research project on the epidemiology and care of individuals with CLP in South Africa [41].

## Methodology

### Conceptual framework

The Registered Nurses’ Association of Ontario (RNAO) developed an IPC framework, entitled *Interprofessional Competency Framework Self-Assessment Tool (ICFSAT)* that describes the competencies required for collaborative practice in health-care teams [25]. The development methodology included a systematic review of the IPC literature, and deliberations by an expert panel and advisory group [25]. The framework consists of a self-administered questionnaire that allows individual health professionals to reflect on their areas of strength in collaborative practice and those areas that need improvement [25]. The intention of the ICFSAT is for health professions to use the self-assessment to enhance IPC, thereby improving patient, organisational, and system outcomes [25].

The ICFSAT of the RNAO consists of seven competency domains and 51 items (Table 1) that measure the knowledge, skills, attitudes and values essential for IPC [25]. All items are assessed on a 4-point Likert scale of 4 = always, 3 = sometimes, 2 = rarely, and 1 = never. There is also an option of “does not apply” which is not rated [25].

**Table 1:** Summary of interprofessional competency framework self-assessment tool

Domain	Description
*Care expertise *(8 items)	Inter-disciplinary care requires collaboration between health professionals and patients and their families and circle of care in order to identify and take advantage of each person’s care expertise
*Shared power *(4 items)	Willingness to share power as a commitment to create balanced relationship through democratic practices of leadership, decision-making, authority and responsibility
*Collaborative leadership *(10 items)	Collaborative leadership (also called reciprocal or shared leadership) is a people—and relationship—focused approach based on the premise that answers should be found in the collective (the team)
*Shared decision-making *(2 items)	Shared decision-making gives all team members, including patients, the opportunity to contribute their knowledge and expertise, to arrive collaboratively at an optimal goal
*Optimizing professional role and scope* (10 items)	Exemplary inter-disciplinary care let all team members work to their full scope of practice and takes advantage of the synergies professionals working together can create
*Effective group function *(9 items)	A health-care system that supports effective teamwork can improve the quality of patient care, enhance patient safety, and reduce workload issues that cause burnout among professionals
*Competent communication *(8 items)	Competent communication—openness, honesty, respect for each other’s opinions and effective communication skills—is a part of all domains of inter-disciplinary practice

Source: Interprofessional Competency Framework Self-Assessment Tool of the RNAO [25]

Although the ICFSAT of the RNAO [25] was developed for the Canadian context, we selected the framework because it has good face validity, the questionnaire is available free-of-charge, and it includes elements that measure the relationships between health professionals in the team and patients. The framework is also more applicable to a general health-care setting, such as CLP care, compared to some of the other frameworks that were developed in critical care settings [23, 26, 27].

### Study setting and design

We restricted the study to the public health sector in South Africa, as it provides health-care services to the majority (84%) of the population in the country [42]. The study setting consisted of all 11 CLP care centres situated in six of South Africa’s nine provinces that provide specialised care to individuals with CLP.

We used a cross-sectional study design, with the IPC survey completed at all CLP centres during 2016.

### Study population and selection

The study population consisted of all health professionals who were members of the CLP teams at the 11 specialised centres. At each of the centres, the principal investigator (PH) approached all the health professionals who were present on the day of the clinic for CLP care, explained the study verbally, handed each person an information sheet and invited these health professionals to participate in the survey.

### Development of the IPC questionnaire

The study used ICFSAT of the RNAO [25]. We used the framework in its original form, but added a section on background and demographic information to obtain information on gender, age, health professional category (e.g. doctor, nurse, etc.), any specific qualification on CLP care, and continuous professional development (Additional file 1).

### Data collection

Prior to data collection, the questionnaire was piloted with ten members of a CLP team from a newly established centre excluded from the main study. The aim of the pilot was to determine clarity of questions and the time taken for administration. The questionnaire took an average of 15 min to complete and no changes were deemed necessary. The results from the pilot were excluded from the main study.

Following informed consent, each HCP on duty at the CLP care centre, completed a self-administered questionnaire on IPC (Additional file 1). Each questionnaire took around 15 min to complete. The principal researcher conducted quality checks to confirm the completeness of the questionnaires. Data were cleaned and checked for inconsistencies before importing into STATA^®^13 for analysis.

### Data analysis

Cronbach’s alpha coefficients were calculated to determine the reliability and coherence between the 51 items in the seven domains. These ranged from 0.73 to 0.93, indicating high reliability and inter-item correlation (Additional file 2).

In the analysis, a response of “does not apply” was set as a ‘missing’ value in STATA and given a score of zero, which means that it did not influence the mean scores for each domain. We assumed that a score of 4 (always) indicated that IPC for that domain was good, whereas a score of 3 (sometimes), 2 (rarely) or 1 (never) indicated that IPC was sub-optimal, and required improvement.

An overall mean IPC score was computed as well as a mean score for each domain. The responses on each domain were summarised using means, standard deviations, and ranges. In order to examine differences in mean scores, we classified the respondents according to gender, health professional category, and CLP treatment centre. Given the small numbers of health professionals, we allocated two categories. One category called “doctor” included plastic surgeons, maxillo-facial surgeons, orthodontists, paediatricians and dentists. A second category called “therapist” included speech therapists, geneticists, nurses, psychologists and social workers. We used ANOVA to analyse the differences in scores across the domains. All statistical tests were conducted at 5% significance level.

## Results

### Participants’ characteristics

The participants’ characteristics are shown in Table 2. A total of 52 participants completed the questionnaire. The mean age of participants was 41.9 years (range 22–72) and the median was 40 years (inter-quartile range (IQR) 31.5–53). The majority were women (52%). The majority of participants were plastic surgeons (38.5%) followed by speech therapists (23.1%), nurses (9.6%), geneticists (7.7%), orthodontists (5.8%), maxillo-facial surgeons (3.9%). psychologists (3.9%), paediatricians (3.9%), dentist (1.9%) and social workers (1.9%). The health professionals at each CLP centre ranged from two to eight. Three participants (5.8%) reported to have a specific qualification on CLP and all reported participation in continuous professional development.

**Table 2:** Descriptive characteristics of the participants

Characteristics	*n* (%)
Mean age in years (SD)	41.9 (1.8)
Median age in years (IQR)	40 (31.5—53)
Age range in years	22 – 72
Gender	
Male	25 (48.1%)
Female	27 (51.9%)

Health professionals category	*n = 52*
Doctor	
Plastic surgeon	20 (38.5%)
Maxillo-facial surgeon	2 (3.9%)
Orthodontist	3 (5.8%)
Paediatrician	2 (3.9%)
Dentist	1 (1.9%)
Therapist	
Speech therapist	12 (23.1%)
Geneticist	4 (7.7%)
Nurse	5 (9.6%)
Psychologist	2 (3.9%)
Social worker	1 (1.9%)

Number of health professionals per CLP Centre	*n = 52*
SITE 1 (GP)	4 (7.7%)
SITE 2 (GP)	5 (9.6%)
SITE 3 (GP)	3 (5.8%)
SITE 4 (GP)	5 (9.6%)
SITE 5 (GP)	8 (15.4%)
SITE 6 (WC)	6 (11.5%)
SITE 7 (WC)	6 (11.5%)
SITE 8 (KZN)	5 (9.6%)
SITE 9 (FS)	2 (3.9%)
SITE 10 (EC)	5 (9.6%)
SITE 11 (LP)	3 (5.8%)

*GP* Gauteng Province, *WC* Western Cape Province, *KZN* KwaZulu Natal Province, *FS* Free State Province, *EC* Eastern Cape Province, *LP* Limpopo Province

### Mean scores for IPC domains

Table 3 shows the mean scores for each of the seven domains. The highest mean score of 2.92 was obtained for care expertise, whereas effective group functioning obtained the lowest score of 2.55. None of the domains obtained a mean score of 4 (i.e. always), suggesting that they did not collaborate fully as a CLP team and that IPC required improvement.

**Table 3:** Mean scores for IPC domains

Domain	Mean	Standard deviations	Cronbach-α
Care expertise (8 items)	2.92	0.37	0.83
Shared power (4 items)	2.67	0.50	0.88
Collaborative leadership (10 items)	2.72	0.45	0.92
Optimizing professional role and scope (10 items)	2.70	0.46	0.93
Shared decision-making (2 items)	2.69	0.50	0.73
Effective group function (9 items)	2.55	0.50	0.92
Competent communication (8 items)	2.63	0.44	0.87

### Mean IPC domain scores by explanatory factor

Table 4 shows the summary of mean scores by explanatory factor. The mean IPC scores by domain did not differ by gender. None of the scores differed between males and females. The professional category called “doctor” scored higher overall. Compared to the “therapist”, the “doctor” category was more likely to obtain higher mean scores for shared power (*p* < 0.01), collaborative leadership (*p* < 0.04), optimising professional role and scope (*p* < 0.03), effective group function (*p* < 0.01), competent communication (*p* < 0.04) and overall (*p* < 0.02). The mean scores on the domains of care expertise (*p* < 0.0005) and shared power (*p* < 0.01) differed across the CLP care centres.

**Table 4:** Mean IPC domain scores by explanatory factor

Explanatory levels		Care expertise	Shared power	Collective leadership	Shared decision	Profession, role and scope	Group function	Communication	Overall
Gender	Male	2.95	2.76	2.83	2,72	2.76	2.65	2.71	2.77
	Female	2.89	2.58	2.61	2.67	2.64	2.46	2.57	2.63
Professional category	Doctor	2.98	2.83*	2.88*	2.73	2.83*	2.70*	2.76*	2.81*
	Therapist	2.85	2.48*	2.52*	2.64	2.56*	2.38*	2.5*	2.56*
CLP centres	Site 1 (GP)	2.90*	3*	2.6	2.63	2.33	2.28	2.38	2.59
	Site 2 (GP)	2.38*	2.95*	2.62	2.7	3	2.53	2.38	2.65
	Site 3 (GP)	3.04*	2.83*	2.7	2.83	2.73	2.85	3	2.86
	Site 4 (GP)	2.8*	1.8*	2.22	2.6	2.52	2.24	2.53	2.39
	Site 5 (GP)	3.03*	2.56*	2.71	2.5	2.63	2.63	2.66	2.67
	Site 6 (WC)	3.08*	2.75*	2.85	2.67	2.5	2.43	2.61	2.7
	Site 7 (WC)	2.85*	2.58*	2.71	2.75	2.73	2.44	2.52	2.66
	Site 8 (KZN)	3.25*	2.9*	3.05	2.9	3	3	3	3.06
	Site 9 (FS)	3.1*	2.85*	2.92	2.8	2.86	2.8	2.93	2.89
	Site 10 (EC)	2.25*	2.75*	2.5	2.5	2.8	2.33	2.25	2.48
	Site 11 (LP)	3.08*	2.58*	2.87	2.83	2.73	2.85	2.71	2.75

*Differences statistically significant (*p* < 0.05)

## Discussion

This was one of the first studies to use the Interprofessional Competency Framework Self-Assessment Tool of the RNAO [25] to analyse the IPC among CLP team members at the 11 specialised care centres in the South African public sector. The findings from our study suggest that overall IPC was sub-optimal or needing improvement, as the overall mean score was below 4 (Table 4). We could not find similar studies that used the RNAO’s Interprofessional Competency Framework Self-Assessment Tool or any other IPC framework in CLP care, in order to compare our study findings. Although the domains overlap, the mean scores for each of the seven domains were also sub-optimal and are discussed below.

### Care expertise

The domain of care expertise, which measures the collaboration among HCP, the patient and their families, obtained a mean score of 2.92. There were no differences by gender or health professional category. The differences in mean scores were influenced by CLP centre. Site 2 in Gauteng (GP) Province obtained the lowest mean score of 2.38, while site 8 in KwaZulu-Natal (KZN) obtained the highest mean score of 3.25.

### Shared power

The domain of shared power, which measures the willingness to share power in decision-making, authority and responsibility, obtained a mean score of 2.67. There were no gender differences found on the willingness to share power. The doctors were more likely to report shared power than other categories of health professionals. Other studies have also found that there is a professional hierarchy, with more power invested in physicians who dominate decision-making, and this could be a barrier to IPC [43–45]. Site 1 in GP obtained the highest mean score of 3 while Site 4 in GP obtained the lowest mean score of 1.8.

### Collaborative leadership

In our study, Site 8 in KZN obtained the highest score of 3 for the domain of collaborative or shared leadership whilst Site 4 in GP had the lowest score of 2.2. The doctors were more likely to report collaborative leadership compared to the therapists. Another study has argued for collaborative leadership is necessary to ensure that the work atmosphere supports the health-care team to enable quality care to patients [46].

### Shared decision-making

This domain measured the extent to which all team members, including patients, have the opportunity to contribute their knowledge and expertise, to optimise treatment goals. Joint decision-making enables sharing of knowledge with the other team members and learning from each other for the benefit of patients [47]. Our study findings showed that Site 8 in KZN had the highest score of 2.9 while Site 5 in GP and Site 10 in Eastern Cape (EC) had lowest scores of 2.5. There were no statistically significant differences by gender, professional category and care centres. The mean scores imply that shared decision-making in CLP care requires improvement.

### Optimising profession, role and scope

This domain evaluated the inter-disciplinary model of care that allows all team members to work to their full scope of practice, taking advantage of the synergies created by professionals working together. Site 2 in GP and Site 8 in KZN obtained the highest scores of 3 for this domain, while Site 1 in Gauteng had the lowest score of 2.3. The doctor category was more likely to report optimising profession, role and care compared to the therapist category. Other studies have found that this inter-disciplinary model of care involving physicians, pharmacists and nurses improved the management of hypertension in individuals with chronic diseases [48, 49]. Our study results highlight the importance of collaboration among members of the multi-disciplinary team to optimise health care provision to individuals with CLP. Possible strategies include joint continuing professional development sessions on evidence-based CLP management, and institutional support and leadership.

### Effective group functioning

This domain measures the extent to which the health-care system supports effective teamwork. Site 8 in KZN obtained the highest mean score of 3 while Site 4 in GP had a lowest score at 2.2. There were statistically significant differences by professional category, with the doctors more likely to report effective group function compared to the therapists.

### Competent communication

This domain focuses on openness, honesty, respect for each other’s opinions and effective communication skills. Communication is important to convey consistent messages that improves patient care. Site 3 in GP and Site 8 in KZN had the highest scores of 3 while Site 4 in GP obtained the lowest score of 2.2. The doctors were more likely to report higher scores on competent communication compared to the therapists.

A study in Nigeria found that an insufficient number of health professionals and sociocultural issues hindered IPC among health-care team members responsible for CLP [50]. The staff numbers found in our study were also small compared to staff numbers of CLP centres reported in other parts of Africa [51], Brazil [52], China [53], United Kingdom [54] and USA [55]. Hence, these small numbers influenced the scores.

Our study findings have implications for the treatment and care of individuals with CLP. The study revealed that the professional category, and to a lesser extent CLP centre, explained the differences in mean scores. In the majority of domains (shared power, collective leadership, optimizing profession role and scope, effective group function and effective communication), doctors obtained higher mean scores compared to therapists. Other studies have also found that doctors tend to dominate, both because of their training, professional status and socialisation [43, 56]. The complexity of the CLP condition and the long-term nature of treatment of individuals require a multi-disciplinary team that practise IPC. The seven domains of the RNAO could be used to guide practical strategies to enhance IPC in the 11 CLP centres, starting with those that do not require additional resources. However, IPC requires institutional support and leadership, as well as mentoring and coaching to unlearn certain behaviours [5], 57. Other strategies to enhance IPC include inter-disciplinary rounds and clinical discussions, multi-disciplinary meeting or video conferencing on patient management [47, 58].

## Limitations and strengths of the study

Although the study was limited by the small sample size, we obtained high response rates among the health professionals involved in CLP care at each of the centres. Hence, we captured the universe of health professionals at each centre. The cross-sectional nature of the study means that we obtained the perspectives of health professionals at a point in time, using a scoring system. Further research is needed to determine the qualitative reasons for the differences in the scores at the various specialised centres. The potential social desirability bias was minimised by using a validated instrument that was self-administered [59, 60].

The Interprofessional Competency Framework Self-Assessment Tool of the RNAO [25] was developed in Canada, and has not been validated formally in other geographical settings. In addition, Likert scales may be interpreted differently or be influenced by culture and/or geographical locations [61, 62]. However, the pilot study found that the questionnaire was well understood by the health professionals at the CLP centre excluded from the main study. Nonetheless, a formal validation of the Interprofessional Competency Framework of the RNAO is an area for further research, as well as its application among a larger sample of health professionals and in different health-care settings.

There are several strengths of our study, which include the measurement of IPC using a validated instrument, obtaining baseline IPC data at all the CLP centres in the South African public sector, and initiating the discourse on IPC in the treatment and care of individuals with CLP.

## Conclusions

IPC in CLP treatment and care has huge potential to enhance patient outcomes and quality of care. The seven IPC categories could be used as a guide to develop specific strategies to enhance IPC among CLP teams. The study findings can be used as a foundation for improving communication and teamwork in CLP care, and promoting the coordination of complex care processes across the lifespan of all individuals with CLP.

## Supplementary Information

**Additional file 1.** CLP Team IPC Questionnaire.

**Additional file 2.** Study data set.

## Abbreviations

CLP: Cleft lip and palate
EC: Eastern Cape Province
FS: Free State Province
GP: Gauteng Province
HREC: Human Research Ethics Committee
IPC: Interprofessional collaboration
ICFSAT: Interprofessional Competency Framework Self-Assessment Tool
IQR: Inter-quartile range
KZN: KwaZulu Natal Province
LP: Limpopo Province
LR: Laetitia Rispel
MDT: Multi-disciplinary team
PH: Phumzile Hlongwa
PhD: Doctor of Philosophy
RNAO: Registered Nurses’ Association of Ontario
USA: United States of America
WC: Western Cape Province
WHO: World Health Organization
