# Supplementary material for: Interprofessional collaboration among health professionals in cleft lip and palate treatment and care in the public health sector of South Africa
Source: Hum Resour Health. 2021 Feb 27;19:25. doi: 10.1186/s12960-021-00566-3 (PMC7912817; doi:10.1186/s12960-021-00566-3)
Supplement: Supplementary file 1 — Additional file 1. CLP Team IPC Questionnaire. [file 12960_2021_566_MOESM1_ESM.pdf]

Additional File 1 CLP Team IPC Questionnaire

Examining inter-professional collaboration among cleft lip and palate healthcare team members in the public health sector of South Africa

FOR OFFICIAL USE ONLY

1. Participant number ☐☐☐☐

2. Study site
   - ☐ 1
   - ☐ 2
   - ☐ 3
   - ☐ 4
   - ☐ 5
   - ☐ 6
   - ☐ 7
   - ☐ 8
   - ☐ 9
   - ☐ 10
   - ☐ 11

   ☐☐☐☐☐☐

3. Date of interview DD/MM/YY ☐☐☐☐☐☐

STATEMENT OF CONSENT

I have been given an information sheet and I understand the objectives of the study.

I further understand that my responses will be kept confidential and that it is up me whether or not to complete this questionnaire.

It has been explained to me that even if I choose not to complete this questionnaire, I should still return the questionnaire to the researchers and indicate **No** in the space below.

My refusal to participate will in no way prejudice me**.**

I agree voluntarily to complete the questionnaire **(please tick).**

☐ **Yes**

☐ **No**

SECTION 1 – BACKGROUND INFORMATION

OFFICE USE ONLY

□ 101. What is your gender?

□Male (0)
□Female (1)

□ 102. What is your age in years?……………………………………………………

□ 103. Which of the following groups is your profession?

□Geneticist/Genetic Counsellor(1)
□Plastic Surgeon(2)
□Orthodontist(3)
□ENT Surgeon(4)
□Maxillo-Facial Surgeon(5)
□Paediatric Dentist(6)
□Paediatric surgeon(7)
□Speech therapist(8)
□Other, please specify(9)…………………………………………………

□ 104. Do you hold any formal qualification on CLP care?

□ No… 0
□ Yes… 1

□ 105. Do you participate on continuous professional development courses?

□ No… 0
□ Yes… 1

SECTION 2 – CARE EXPERTISE						
	Inter-disciplinary care requires collaboration between health –care professionals and patients and their families and circle of care in order to identify and take advantage of each person's care expertise. To support inter-disciplinary practice, practitioners are able to:	Never (1)	Rarely (2)	Sometimes(3)	Always(4)	Does'nt apply (0)
201.	Support the participation of patients/clients, their families, and / or community representatives as integral partners alongside health-care personnel					
202.	Share information with patients/clients (or family and the community) in a respectful manner and in such a way that it is understandable, encourages discussion and enhances participation in decision making					
203.	Ensure that appropriate education and support is provided to patients/clients, family members and others involved with care or service					
204.	Listen respectively to the expressed needs of all parties in shaping and delivering care or services					
205.	Conduct a collaborative inter-professional assessment to identify what expertise is required and then individualize for each patient/client					

206.	Coordinated effort to find the best expert for the patient/client					
207.	Patients/clients are full participants in their own care					
208.	Include specific contributions and collective knowledge as dictated by the complexity of the patients/clients needs					
**SECTION 3 – SHARED POWER**						
	Willingness to share power is a commitment to create balanced relationship through democratic practices of leadership, decision making, authority and responsibility. To support inter-personal practice, practitioners are able to:	Never (1)	Rarely (2)	Sometimes(3)	Always(4)	Does not apply (0)
301.	Leverage opportunities for all team members to contribute					
302.	Create balanced power relationships					
303.	Establish a safe environment to express diverse opinions					
304.	Consider points of view of all care providers					
**SECTION 4 – COLLABORATIVE LEADERSHIP**						
	Collaborative leadership (also called reciprocal or shared leadership) is a people – and relationship – focused approach based on the premise that answers should be found in the collective (the team). To support inter-disciplinary practice, practitioners collaboratively determine who will provide group leadership in any given situation by supporting:	Never (1)	Rarely (2)	Sometimes(3)	Always(4)	Does not apply (0)
401.	Work with others to enable effective patients/client outcomes					
402.	Advance interdependent working relationships among all participants					
403.	Facilitation of effective team processes					
404.	Establish a climate for collaborative practice among all participants					
405.	Co-create a climate for shared leadership and collaborative practice					
406.	Apply collaborative decision making principles					
407.	Integrate the principles of continuous quality improvement to work processes and outcomes					
408.	Share accountability that addresses power and hierarchy					
409.	Utilize structures and processes to advance exemplary care					
410.	Work with others to enable effective patients/client outcomes					

SECTION 5 – SHARED DECISION MAKING						
	Shared decision-making gives all team members, including patients, the opportunity to contribute their knowledge and expertise, to arrive collaboratively at an optimal goal. To support inter-disciplinary practice, /practitioners are able to:	Never (1)	Rarely (2)	Sometimes(3)	Always(4)	Does not apply (0)
501.	Recognize and respect each other's knowledge and expertise, regardless of occupation and formal position					
502.	Willing to accept responsibility for decisions					

SECTION 6 – OPTIMIZING PROFESSION, ROLE AND SCOPE						
	Exemplary inter-disciplinary care let all team members work to their full scope of practice and takes advantage of the synergies professionals working together can create. To support inter-disciplinary practice, practitioners are able to:	Never (1)	Rarely (2)	Sometimes(3)	Always(4)	Does not apply (0)
601.	Describe their role and others'					
602.	Recognize and respect the diversity of other health and social care roles, responsibilities, and competencies					
603.	Perform their own roles in a culturally respectful way					
604.	Communicate roles, knowledge, skills, and attitudes using appropriate language					
605.	Consider the roles of others in determining own professional roles					
606.	Access others' skills and knowledge appropriately through consultation					
607.	Consider the roles of other in determining own professional and inter-professional roles					
608.	Integrate competencies/roles seamlessly into models of service delivery					
609.	Demonstrate knowledge application of own profession/roles/scope					
610.	Explore and integrate roles of others					

SECTION 7 – EFFECTIVE GROUP FUNCTION						
	A health-care system that supports effective teamwork can improve the quality of patient care, enhance patient safety, and reduce workload issues that cause burnout among professionals. To support inter-disciplinary practice, practitioners are able to:	Never (1)	Rarely (2)	Sometimes(3)	Always(4)	Does not apply (0)
701.	Understand the process of team development					
702.	Develop a set of principles for working together that respects the ethical values of members					
703.	Effectively facilitate discussions and interactions among team members					
704.	Participate, and be respectful of all members' participating , in collaborative decision making					
705.	Regularly reflect on their functioning with team learners/practitioners and patients/clients families					

706.	Establish and maintains effective and healthy working relationships with learners/practitioners, patients/clients, and families, whether or not a formalized team exists					
707.	Respect team ethics, including confidentiality, resource and allocation, and professionalism					
708.	Collaborate and engage together to formulate implement and evaluate care					
709.	Assess, Practise and reflect upon effective group processes					
**SECTION 8 - COMPETENT COMMUNICATION**						
	Competent communication – openness, honesty, respect for each other's opinions and effective communication skills – is a part of all domains of inter-disciplinary practice. To support inter-disciplinary, practitioners are able to:	Never (1)	Rarely (2)	Sometimes(3)	Always(4)	Does not apply (0)
801.	Establish team work communication principles					
802.	Actively listen to other team members including patients/ clients/families					
803.	Communicate to ensure common understanding of care decisions					
804.	Develop trusting relationships with patients/clients/families and team members					
805.	Effectively use information and communication technology to improve inter-professional patient/client/community-centered care					
806.	Is clear, focused, transparent and respectful					
807.	Constructively manages conflict					
808.	Maintains and enhances the relationship					

Thank you for participating
