# Supplementary material for: Interprofessional collaboration among health professionals in cleft lip and palate treatment and care in the public health sector of South Africa
Source: Hum Resour Health. 2021 Feb 27;19:25. doi: 10.1186/s12960-021-00566-3 (PMC7912817; doi:10.1186/s12960-021-00566-3)
Supplement: Supplementary file 2 — Additional file 2. Study data set. [file 12960_2021_566_MOESM2_ESM.pdf]

Study site	gender	age	profession	Doctor
2	1	30	7	Peads
2	1	35	9	
2	0	50	1	
2	1	42	2	Plastic
2	0	55	2	Plastic
9	0	36	2	Plastic
9	0	56	2	Plastic
11	1	33	2	Plastic
11	0	56	2	Plastic
11	0	51	7	Paed
3	0	35	2	Plastic
3	0	42	2	Plastic
3	0	37	2	Plastic
4	1	27	8	
4	1	22	8	
4	1	24	8	
4	1	29	8	
4	1	26	8	
7	0	48	2	Plastic
7	1	59	9	
7	1	72	10	
7	1	59	8	
7	0	37	2	Plastic
7	1	56	3	ortho
6	0	50	2	Plastic
6	1	42	2	Plastic
6	0	52	2	Plastic
6	1	27	8	
6	0	32	2	Plastic
6	0	59	5	mfos
8	0	58	2	Plastic
8	0	25	6	dentist
8	1	37	1	
8	0	40	5	mfos
8	1	36	2	Plastic
10	1	34	2	Plastic
10	0	40	2	Plastic
10	0	62	2	Plastic
10	0	32	2	Plastic
10	1	26	8	
5	1	48	10	
5	1	31	8	
5	1	34	8	
5	0	44	10	
5	0	54	3	ortho
5	1	59	10	
5	1	64	10	
5	0	46	5	mfos
1	1	24	8	

1	0	42	8	
1	1	31	2	Plastic
1	1	30	11	

OTHER	clp qualification	cpd	201	202	203
	0	1	2	2	2
Psychologist	0	1	2	2	2
Geneticist	0	1	2	2	2
	0	1	2	2	2
	0	1	2	2	2
	0	1	2	2	2
	0	1	2	2	2
	0	1	3	3	3
	0	1	3	3	3
	0	1	2	3	3
	0	0	2	3	3
	0	1	2	3	3
	0	1	3	3	3
Speech therapist	0	1	3	3	3
Speech therapist	0	1	3	3	3
Speech therapist	0	1	2	3	3
Speech therapist	0	1	3	3	3
Speech therapist	0	1	3	3	3
	0	1	2	3	3
Psychologist	0	1	2	3	3
Nurse	0	0	2	1	2
Speech therapist	0	1	3	3	3
	0	1	3	2	3
	0	1	3	3	3
	0	1	3	3	3
	0	1	3	3	3
	0	1	3	3	3
Speech therapist	0	1	3	3	3
	0	1	2	3	3
	0	1	2	3	3
	0	1	3	3	3
	0	1	3	3	3
Geneticist	0	1	3	3	3
	0	1	3	3	3
	0	1	3	3	3
	0	1	3	3	2
	0	1	3	3	3
	0	1	3	3	3
	0	1	3	3	3
Speech therapist	0	1	2	3	3
Nurse	0	0	2	3	2
Speech therapist	0	1	3	2	2
Speech therapist	0	1	3	3	3
Nurse	0	0	3	3	3
	0	1	3	3	3
Nurse	0	1	3	3	2
Nurse	0	1	3	3	3
	0	1	3	3	3
Speech therapist	0	1	2	2	3

Speech therapist	0	1	3	3	3
	0	1	3	3	3
Social worker	0	1	2	2	3

204	205	206	207	208	301	302	303	304	401
2	2	2	1	2	3	2	3	3	3
2	2	2	1	2	3	2	3	3	3
2	2	2	1	2	3	2	3	3	3
2	2	2	1	2	3	2	3	3	3
2	2	2	1	2	3	2	3	3	3
2	2	2	1	2	3	2	3	3	3
2	2	2	1	2	3	2	3	3	3
3	3	3	3	3	3	3	3	3	3
3	2	3	2	2	3	2	3	3	3
2	3	3	2	2	2	1	3	2	2
3	2	2	2	3	3	2	3	3	2
3	2	2	2	3	3	2	3	3	2
3	3	3	3	3	3	3	3	3	3
3	2	2	3	3	2	2	3	3	2
3	3	3	2	3	2	2	2	2	3
3	1	2	3	2	1	1	0	1	2
3	1	2	1	2	2	2	1	2	3
3	2	2	3	3	2	2	3	3	3
3	3	3	2	3	2	2	2	2	3
3	1	2	2	2	3	2	3	2	3
3	3	3	3	3	2	3	3	2	3
3	3	3	3	3	3	3	3	3	3
3	2	2	2	3	2	3	2	3	2
3	3	3	2	2	3	3	3	3	3
3	3	3	3	3	3	3	3	3	3
3	3	3	2	3	3	2	3	3	3
2	2	2	3	3	3	2	3	3	3
3	3	3	3	3	2	2	3	3	3
3	3	3	2	3	3	2	3	3	3
3	3	3	2	3	3	2	3	3	3
3	3	3	3	3	3	3	3	3	3
3	3	3	3	3	3	2	3	3	3
3	3	3	3	3	3	3	3	3	3
3	3	3	3	3	3	3	3	3	3
3	3	3	3	3	2	3	3	3	3
3	3	3	3	3	3	3	2	3	3
3	3	3	2	3	3	3	3	3	3
3	3	3	2	3	3	3	3	3	3
3	3	3	2	3	3	3	3	3	3
3	2	3	3	3	3	3	2	2	3
3	2	3	3	2	0	1	2	0	1
3	2	2	2	2	2	2	2	2	2
3	3	3	3	3	3	3	3	3	3
3	3	3	3	2	2	2	3	2	3
3	3	3	3	3	3	3	3	3	3
3	3	3	2	2	3	3	3	3	3
3	3	3	3	3	3	3	3	3	3
3	3	3	3	3	3	3	3	3	3
3	2	3	2	2	3	3	3	3	3

3	3	3	3	3	3	3	3	3	3
3	3	3	3	3	3	3	3	3	3
3	2	3	2	2	3	3	3	3	3

402	403	404	405	406	407	408	409	410
2	2	3	3	2	2	2	3	2
2	2	3	3	2	2	2	3	2
2	2	3	3	2	2	2	3	2
2	2	3	3	2	2	2	3	2
2	2	3	3	2	2	2	3	2
2	2	3	3	2	2	2	3	2
2	2	3	3	2	2	2	3	2
3	3	3	3	3	3	3	3	3
3	3	3	2	2	3	3	3	3
3	3	2	1	2	3	2	2	3
2	3	3	2	2	3	3	3	2
3	3	3	2	2	3	3	3	2
3	3	2	3	2	3	2	3	3
3	3	3	3	3	2	3	3	3
2	3	2	2	2	3	2	2	3
2	1	1	1	1	2	1	1	1
2	2	2	2	2	2	1	2	2
3	3	3	3	3	3	3	3	3
3	3	3	3	2	2	3	3	3
3	2	2	2	1	2	1	2	3
3	2	3	2	2	3	2	3	3
3	3	3	3	3	3	3	3	3
2	2	3	2	2	3	2	2	3
3	3	3	3	3	3	3	3	3
3	3	3	3	3	3	3	3	3
3	3	3	2	2	2	2	3	3
3	3	2	2	2	2	2	3	3
2	2	3	3	3	3	3	3	3
3	3	3	2	3	3	3	3	2
3	3	3	2	3	3	3	3	2
3	3	3	3	3	3	3	3	3
3	3	3	3	3	3	3	3	3
3	3	3	3	3	3	3	3	3
3	3	3	3	3	3	3	3	3
3	3	3	3	3	3	3	2	3
3	2	2	3	2	3	3	3	2
3	3	3	3	3	3	3	3	3
3	3	3	3	3	3	3	3	3
3	3	3	3	3	3	3	3	3
2	3	2	2	2	3	2	3	3
1	1	1	2	2	2	0	2	2
2	2	2	2	2	2	2	2	2
3	3	3	3	3	3	3	3	3
3	3	3	2	2	3	3	2	3
3	3	3	3	3	3	3	3	3
3	3	3	3	3	3	3	3	3
3	3	3	2	2	3	3	2	3
3	3	3	3	3	3	3	3	3
3	2	2	2	2	1	1	1	2

3	3	3	3	3	3	3	3	3
3	3	3	3	3	3	3	3	3
3	3	2	2	2	2	1	1	2

501	502	601	602	603	604	605	606	607
2	3	3	3	3	3	2	3	2
2	3	3	3	3	3	2	3	2
2	3	3	3	3	3	2	3	2
2	3	3	3	3	3	2	3	2
2	3	3	3	3	3	2	3	2
2	3	3	3	3	3	2	3	2
2	3	3	3	3	3	2	3	2
3	3	3	3	3	3	3	3	3
3	3	3	3	3	3	3	3	3
2	2	3	2	1	3	3	3	2
3	3	2	3	3	3	2	3	4
3	3	2	3	3	3	2	3	4
2	3	3	3	2	3	2	3	3
3	3	3	3	3	3	3	3	3
3	3	3	3	2	2	3	3	3
3	3	3	3	3	3	3	2	2
2	2	3	3	3	2	2	1	2
3	3	3	3	3	3	3	3	3
2	2	2	2	2	3	3	3	3
3	3	3	3	2	2	3	2	3
3	3	3	3	3	3	3	3	3
3	3	3	3	3	3	3	3	3
3	2	2	2	2	3	2	2	3
3	3	3	3	3	3	3	3	3
3	3	3	3	3	3	3	3	3
3	3	3	3	2	2	3	3	3
3	3	3	3	3	3	2	3	2
3	3	3	3	3	2	2	3	2
2	2	2	2	2	2	2	2	2
2	2	2	2	2	2	2	2	2
3	3	3	3	3	3	3	3	3
3	3	3	3	3	3	3	3	3
3	3	3	3	3	3	3	3	3
3	3	3	3	3	3	3	3	3
3	3	3	3	3	3	3	3	3
2	2	3	3	2	2	3	3	3
3	3	3	3	3	3	3	3	3
3	3	3	3	3	3	3	3	3
3	3	3	3	2	2	3	3	3
3	3	3	3	3	2	3	3	2
0	1	1	0	0	1	1	0	1
2	2	2	2	2	2	2	2	2
3	3	3	3	3	3	3	3	3
3	2	1	3	3	3	3	3	3
3	3	3	3	3	3	3	3	3
3	3	3	3	3	3	3	3	3
3	2	3	3	3	2	3	3	3
3	3	3	3	3	3	3	3	3
2	3	2	2	2	1	1	2	2

3	3	2	3	3	3	3	3	3
3	3	3	3	3	3	3	3	3
2	2	2	2	2	1	1	2	2

608	609	610	701	702	703	704	705	706	707
3	3	3	2	3	3	3	2	2	2
3	3	3	2	3	3	3	2	2	2
3	3	3	2	3	3	3	2	2	2
3	3	3	2	3	3	3	2	2	2
3	3	3	2	3	3	3	2	2	2
3	3	3	2	3	3	3	2	2	2
3	3	3	2	3	3	3	2	2	2
3	3	3	3	3	3	3	3	3	3
2	3	3	3	2	3	3	2	2	3
2	3	1	3	1	3	3	1	1	2
3	2	2	2	3	3	3	3	2	3
3	2	2		3	3	3	3	2	3
3	3	3	3	3	3	3	3	2	3
3	3	3	3	3	3	3	3	3	3
2	3	2	2	3	2	3	2	3	2
2	2	2	3	3	1	2	2	3	3
2	3	2	2	1	2	2	1	2	2
3	3	3	3	3	3	3	3	3	3
3	3	3	3	2	3	2	2	1	3
2	2	2	1	2	1	3	1	2	2
3	3	2	3	3	3	3	3	3	3
3	3	3	3	3	2	2	2	3	3
3	3	3	2	2	2	2	2	2	3
3	3	2	3	3	3	3	3	3	3
3	3	3	3	3	3	3	3	3	3
3	3	3	3	3	3	3	1	3	2
3	3	2	2	3	3	3	2	3	3
2	2	3	2	1	2	2	1	3	3
2	2	2	2	2	2	2	2	3	3
2	2	2	2	2	2	2	2	3	3
3	3	3	3	3	3	3	3	3	3
3	3	3	3	3	3	3	3	3	3
3	3	3	3	3	3	3	3	3	3
3	3	3	3	3	3	3	3	3	3
3	3	3	3	3	3	3	3	3	3
3	3	3	3	2	3	2	2	2	3
3	3	3	3	3	3	3	3	3	3
3	3	3	3	3	3	3	3	3	3
3	3	3	3	3	3	3	3	3	3
2	3	3	3	3	3	3	2	2	3
1	2	2	2	1	0	0	1	2	3
2	2	2	2	2	2	2	2	2	2
3	3	3	3	3	3	3	3	3	3
4	3	3	3	3	3	2	3	3	3
3	3	3	3	3	3	3	3	3	3
3	3	3	3	3	3	3	3	3	3
3	3	3	3	3	2	3	3	3	3
3	3	3	3	3	3	3	3	3	3
1	2	2	2	1	2	2	1	2	2

3	3	3	2	3	3	3	3	3	3
3	3	3	3	3	3	3	3	3	3
1	2	2	2	2	2	2	1	2	2

708	709	801	802	803	804	805	806	807	808
2	2	2	3	2	1	1	3	3	2
2	2	2	3	2	1	1	3	3	2
2	2	2	3	2	1	1	3	3	2
2	2	2	3	2	1	1	3	3	2
2	2	2	3	2	1	1	3	3	2
2	2	2	3	2	1	1	3	3	2
2	2	2	3	2	1	1	3	3	2
3	3	3	3	3	3	3	3	3	3
2	2	2	3	3	3	2	3	2	2
2	1	2	3	3	2	2	3	2	3
3	3	3	3	3	3	2	3	3	3
3	3	3	3	3	3	2	3	3	3
3	3	3	4	3	4	3	3	3	3
3	3	3	3	3	3	3	3	3	3
2	1	1	3	2	3	3	3	3	3
1	1	2	2	2	3	3	3	3	2
1	1	2	2	2	2	1	3	2	2
3	3	3	3	3	3	3	3	3	3
3	3	2	2	3	2	2	2	2	2
1	1	1	1	1	3	1	2	1	2
3	3	3	3	3	3	3	3	3	3
3	2	2	2	3	3	3	2	2	3
2	2	3	3	3	3	2	2	3	3
3	3	3	3	3	3	3	3	3	3
3	3	3	3	3	3	3	3	3	3
3	1	3	3	3	3	3	3	3	3
3	2	3	3	3	3	2	2	3	3
2	1	2	3	3	3	3	2	3	2
2	2	2	2	3	2	2	2	2	2
2	2	2	2	3	2	2	2	2	2
3	3	3	3	3	3	3	3	3	3
3	3	3	3	3	3	3	3	3	3
3	3	3	3	3	3	3	3	3	3
3	3	3	3	3	3	3	3	3	3
3	3	3	3	3	3	3	3	3	3
3	2	2	3	3	3	3	3	2	3
3	3	3	3	3	3	3	3	3	3
3	3	3	3	3	3	3	3	3	3
3	3	3	3	3	3	3	3	3	3
3	2	3	3	2	3	3	3	3	3
2	1	2	1	2	3	3	2	1	2
2	2	2	2	2	2	2	2	2	2
3	3	3	3	2	3	2	3	3	3
3	2	3	3	3	3	2	3	2	3
3	3	3	3	3	3	3	3	3	3
3	3	3	3	3	3	3	3	3	3
3	2	2	2	2	3	3	3	3	3
3	3	3	3	3	3	3	3	3	3
1	1	2	2	2	1	1	2	2	2

3	3	3	3	3	3	3	3	3	3
3	3	3	3	3	3	3	3	3	3
2	1	2	2	2	1	1	2	2	2
